# Comparative Evolutionary Genomics Reveals Genetic Diversity and Differentiation in *Bacteroides fragilis*

**DOI:** 10.3390/genes15121519

**Published:** 2024-11-27

**Authors:** Yoshinori Muto, Kaori Tanaka

**Affiliations:** 1 Division of Anaerobe Research, Life Science Research Center, Gifu University, Gifu City 501-1194, Gifu, Japan; tanaka.kaori.y7@f.gifu-u.ac.jp; 2 United Graduate School of Drug Discovery and Medical Information Sciences, Gifu University, Gifu City 501-1194, Gifu, Japan; 3 Institute for Glyco-core Research (iGCORE), Gifu University, Gifu City 501-1193, Gifu, Japan

**Keywords:** *Bacteroides fragilis*, pan-genome, Tajima’s D, Hudson’s fixation index (Fst), recombination, *Bacteroides hominis*

## Abstract

Background/Objectives: *Bacteroides fragilis* is the pathogenic anaerobe most commonly isolated from intra-abdominal infections, abscesses, and blood. Despite its clinical importance, research on its pan-genome-scale evolution is still limited. Methods: Herein, we analyzed the pan-genome architecture of 374 *B. fragilis* strains to explore their intra-species genomic diversity and evolutionary patterns. Results: Our analysis revealed an open pan-genome with a high proportion of accessory genomes, indicating high genetic variability. Accessory genome genes were substantially enriched in the functions of “Replication, Recombination, and Repair” suggesting their roles in gene transfer and divergence. Phylogenomic analysis divided *B. fragilis* into two distinct clades: divisions I and II, differing in gene content, antimicrobial resistance genes, and mobile genetic elements. Division II revealed higher Tajima’s D values, suggesting that it separated after *B. fragilis*’s recent species diversification. The extreme shift in the distribution of gene-wise Hudson’s fixation index (Fst) values for each division suggested that several genes are highly differentiated or evolved between the two clades. Average nucleotide identity and 16S rRNA analyses showed that *B. fragilis* division II represents a distinct species, *Bacteroides hominis*. Additionally, a considerable depletion of recombination in genes with Fst values > 0.99 was noted, suggesting that the highest Fst genes with little recombination are the basis for differentiation between divisions. Conclusions: Overall, this study enhances the understanding of *B. fragilis*’s genomic diversity, evolutionary dynamics, and potential role in pathogenesis, shedding light on its adaptation and diversification.

## 1. Introduction

*Bacteroides fragilis* is a Gram-negative anaerobe that plays a key role in supporting immune function and maintaining an anti-inflammatory environment within the intestinal lumen [1]. Despite its lower abundance in fecal isolates [1], it is the most frequently isolated pathogenic anaerobe from intra-abdominal infections, abscesses, and blood [2,3]. *B. fragilis*’s pathogenicity is linked to several virulence factors, including capsular polysaccharides [4], proteases [5], and *B. fragilis* toxin [6]. Resistance to oxidative stress and extreme aerotolerance also contribute to its role in extraintestinal infections [7]. Comparative studies have shown distinct β-lactamase gene profiles between *B. fragilis* and non-*fragilis Bacteroides* species [8], with notable intra-species diversity of *B. fragilis* in antimicrobial resistance genes [8,9,10,11]. While these factors are recognized as important for *B. fragilis*’s pathogenesis, their relative contributions and precise mechanisms remain unclear, warranting further investigation.

The pan-genome represents the entire gene repertoire of a species, divided into the core genome (genes shared by all strains) and accessory genome (dispensable genes not present in all strains) [12]. Comparative genomics of pan-genomes has become a key approach to understanding species traits, environmental adaptation, and speciation [13]. With the rise of whole-genome sequencing of closely related species populations, genomic variation caused by horizontal gene transfer and gene diversification has been widely recognized to play a substantial role in adaptation to distinct ecological niches [14,15]. *B. fragilis*, for instance, is divided into two divisions based on the absence (division I) or presence (division II) of the chromosomally encoded carbapenemase gene cfiA [8,16]. Furthermore, divisions I and II have been previously shown to exhibit differential gene abundances associated with nutrient utilization, niche occupancy, and virulence [17]. Thus, evolutionary mechanisms influencing the pan-genome and genomic diversity can considerably affect *B. fragilis*’s adaptive potential and differentiation [18,19]. However, comprehensive pan-genome-scale evolutionary studies are still required to clarify the mechanism behind its differentiation and pathogenicity.

To better understand the evolutionary dynamics and functional differentiation of *B. fragilis*, we conducted a comprehensive pan-genomic analysis, focusing on intra-species genomic diversity and evolutionary events. Additionally, we focused on the population genetics characterization of pan-genome genes that are differentially shared by the *B. fragilis* genomes. Our findings shed light on the evolutionary mechanisms driving bacterial pathogenicity and highlight the potential functional diversification of genes linked to species differentiation.

## 2. Materials and Methods

### 2.1. Genome Data Set

A total of 616 *B. fragilis* genomes, available as of May 2022, were downloaded from the National Center for Biotechnology Information (NCBI) genome FTP site (https://www.ncbi.nlm.nih.gov/genomes (accessed on 24 May 2022)). Genomes with <95% completeness and >5% contamination were excluded using CheckM v.1.1.3 [20]. Furthermore, whole-genome average nucleotide identity (ANI) was calculated for each pair of genomes using pyani v.0.2.7 [21] in ANIm mode. Finally, assemblies comprising > 100 contigs were excluded. In total, 374 high-quality *B. fragilis* genomes were retained for use in this study. Supplementary Table S1 lists the accession numbers, strain names, and contig numbers. Genomes were annotated using Prokka v.1.12 with default parameters [22].

### 2.2. Pan-Genome and Phylogenetic Analyses

*B. fragilis*’s pan-genome was analyzed using Roary v.3.13.0 [23]. The inputs were the gene feature format (GFF) files generated by Prokka annotations. The software generated a gene presence/absence matrix, identifying core and accessory genes. Sequences of individual gene families were aligned using PRANK v.170427 [24]. Pan-genome and core genome profiles were evaluated and visualized using the Pan-Genome Profile Analyze Tool (PanGP) version 1.0.1 [25]. Furthermore, the distance-guided (DG) algorithm was employed using default parameters. Heap’s law model was used to fit the pan-genome size and openness [12,26].

A concatenated core gene alignment from Roary (as previously described) was used to determine the *B. fragilis* phylogeny. The maximum likelihood tree was constructed using IQtree v.2.2.0.3 [27], with 1000 ultrafast bootstraps, and the GTR+F+I+I+R10 model was selected using ModelFinder [28]. The phylogenetic tree was visualized using the Interactive Tree of Life program [29]. Identification of closely related species and the measurement of their 16S rRNA sequence similarity were carried out using the EzBioCloud database [30]. Pan-genome network analysis utilized Roary’s gene presence/absence matrix. The Jaccard similarity coefficient, based on the number of shared genes relative to the total number of genes across a pair of genomes, was calculated using the GraPPLE script pw_similarity.py [31]. The resulting genome similarity networks were filtered in Graphia [32] using the edge weight and k-nearest neighbor algorithms. Next, the network was clustered using the MCL-Markov algorithm via the Cytoscape plugin ClusterMaker [33] and visualized as a network using Cytoscape v.3.10.0 [34].

### 2.3. Functional Annotation and Classification of Genes

Gene annotation and Clusters of Orthologous Groups (COG) category assignment were performed through a sequence search of the eggNOG database [35] using eggNOG-Mapper [36] with default settings. Antimicrobial resistance (AMR) genes and resistance-associated point mutations were identified using AMRFinderPlus [37], which references the NCBI Bacterial Antimicrobial Resistance Reference Gene Database. Mobile genetic elements (MGEs), including transposons, insertion sequences, and integrative and conjugative elements, were identified using MobileElementFinder v. 1.1.2 [38].

### 2.4. Evolutionary and Population Genomics Analysis

We examined the selective pressure acting on accessory and core genes by calculating the ratio of nonsynonymous to synonymous substitutions (dN/dS) for core and accessory gene alignments using the maximum likelihood implementation in GenomegaMap, which is a phylogeny-free method optimized for calculating within-species dN/dS [39]. Population genetics statistics, including nucleotide diversity (π) and Tajima’s D, were calculated using EggLib v3.0.0 [40]. Gene sequence alignments were obtained using the codon-aware alignment program PRANK, and only gene clusters occurring in ≥5 strains were utilized. Additionally, Hudson’s fixation index (Fst) statistics were calculated using EggLib v3.0.0 for gene alignments comprising ≥5 sequences per lineage [41]. FastGEAR [42] was employed with default parameters to identify lineage-specific recombinant segments (ancestral recombination) and strain-specific recombinant segments (recent recombination) in individual gene alignments.

### 2.5. Statistical Analysis and Data Visualization

Z-test implemented in the StatsModels module of Python was used for determining significant differences in the proportions of COG categories. Box plots and bar graphs were generated using SigmaPlot 14.0. The odds ratios of FST > 0.99 versus FST_all genes for ancestral and recent recombination were calculated using the fisher.test function in R.

## 3. Results and Discussion

### 3.1. General Characteristics of the B. fragilis Genome

In May 2022, all *B. fragilis* genome sequences available from the NCBI Assembly database were downloaded. After quality assessment with CheckM [20], genomes were further filtered based on the number of contigs (see Methods for details). A total of 374 high-quality genomes (completeness > 95%, contamination < 5%) with <100 contigs were identified (Supplementary Table S1).

To analyze the comprehensive genomic profile of the *B. fragilis* strains, we employed Roary [23] to estimate the pan-genome, which represents the aggregate of all genes present in a species’ genome [26]. The 374 *B. fragilis* strains exhibited a pan-genome of 35,277 genes, with a range of 3898–5307 genes per genome (mean = 4402). Of these, 1862 genes were found in at least 95% of the strains and were considered core genes. An additional 39 genes, present in 94–95% of the strains, were designated as the soft core genes. Together, these 1901 genes form the core genome, a small fraction of the pan-genome of the entire species. The number of shell genes present in 15–94% of the strains was 4363, whereas the number of cloud genes present in <15% of the strains was 29,013 (Supplementary Table S2). We designated the shell and cloud genes as the accessory genome, which constitutes a substantial portion of the pan-genome.

### 3.2. Functional Characterization of the B. fragilis Pan-Genome

**Figure 1:**
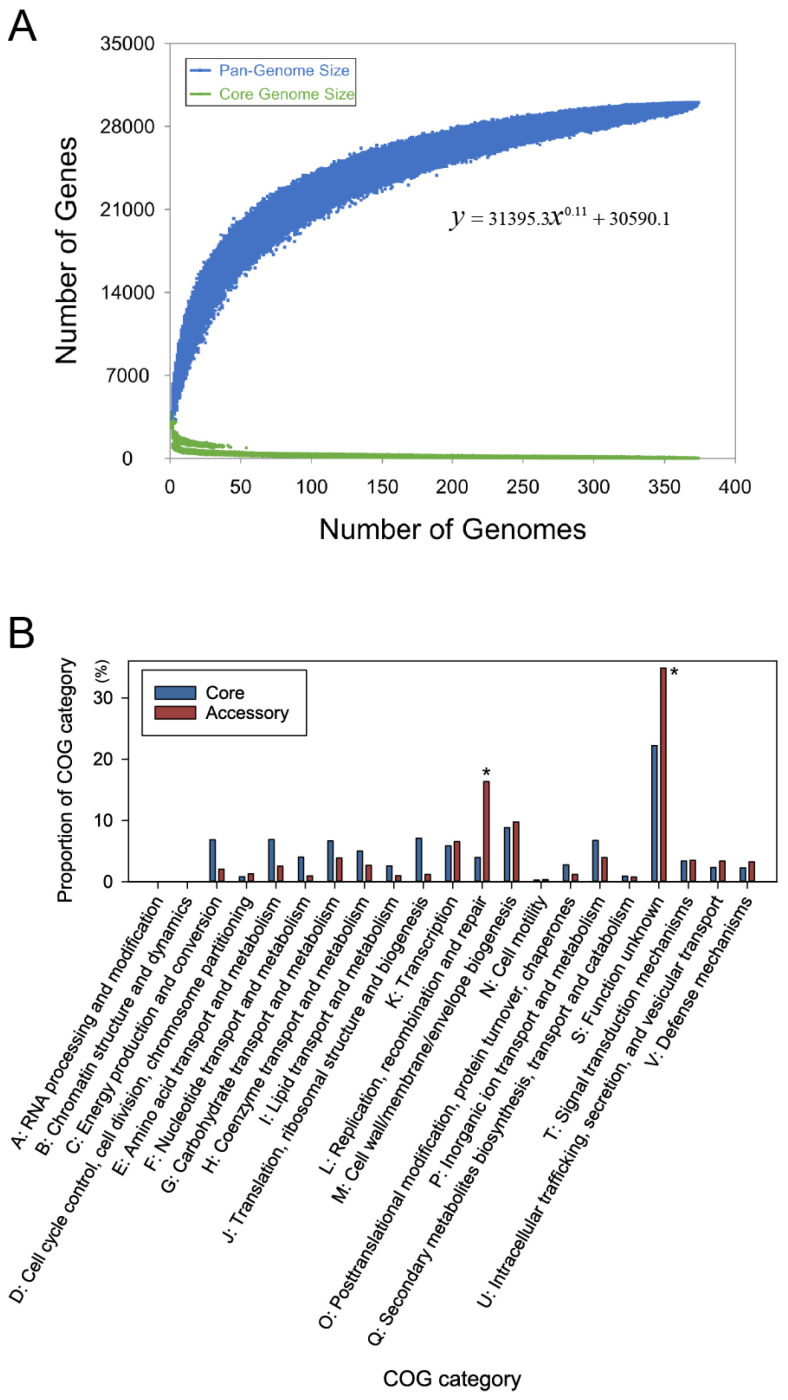
The pan-genome, core genome, and accessory genome profiles of *B. fragilis*. (**A**) Genome size change curves of the pan-genome (blue) and core genome (green) as a function of the genomes added. The formula indicates the least-squares fit of the power law for the average values of the pan-genome. (**B**) Clusters of Orthologous Groups (COG) functional annotation of the *B. fragilis* core and accessory genomes. Asterisks indicate significant enrichment of the accessory genome genes as compared to the core genome in the individual category (*p* < 0.001, Z-test).

The cumulative curve for *the B. fragilis* pan-genome showed no clear plateau (Figure 1A), suggesting that the pan-genome size would continue to expand with more genome numbers. Furthermore, several genes were incorporated into each additional genome (Supplementary Figure S1). Analysis using Heap’s law model [26] confirmed an open pan-genome, with a power-law exponent γ of 0.11 (Figure 1A). In total, 5.4% of the pan-genome was identified as conserved (core genome), whereas the remaining 94.6% exhibited variation across the strains (accessory genome), reflecting high genome variability [12]. These findings are consistent with those of previous studies, which noted that *B. fragilis* has a larger accessory genome proportion than other pathogenic bacteria [17].

The core and accessory genomes were annotated using the COG functional classification database (Figure 1B). The core genomes demonstrated a higher gene proportion within the COG categories related to fundamental biological functions, including “C: Energy production and conversion”, “E: Amino acid transport and metabolism” and “J: Translation, ribosomal structure, and biogenesis” in comparison to the accessory genome. Contrastingly, the accessory genome genes were substantially enriched in a COG category designated “L: The replication, recombination, and repair”, which was significantly more prevalent in the accessory genome than in the core genome (*p* = 3.57 × 10^−32^, Z-test). The COG category genes likely contribute to species diversification, as they are crucial for horizontal gene transfer [43].

### 3.3. Phylogenomic Relationship and Genome Differentiation of B. fragilis

**Figure 2:**
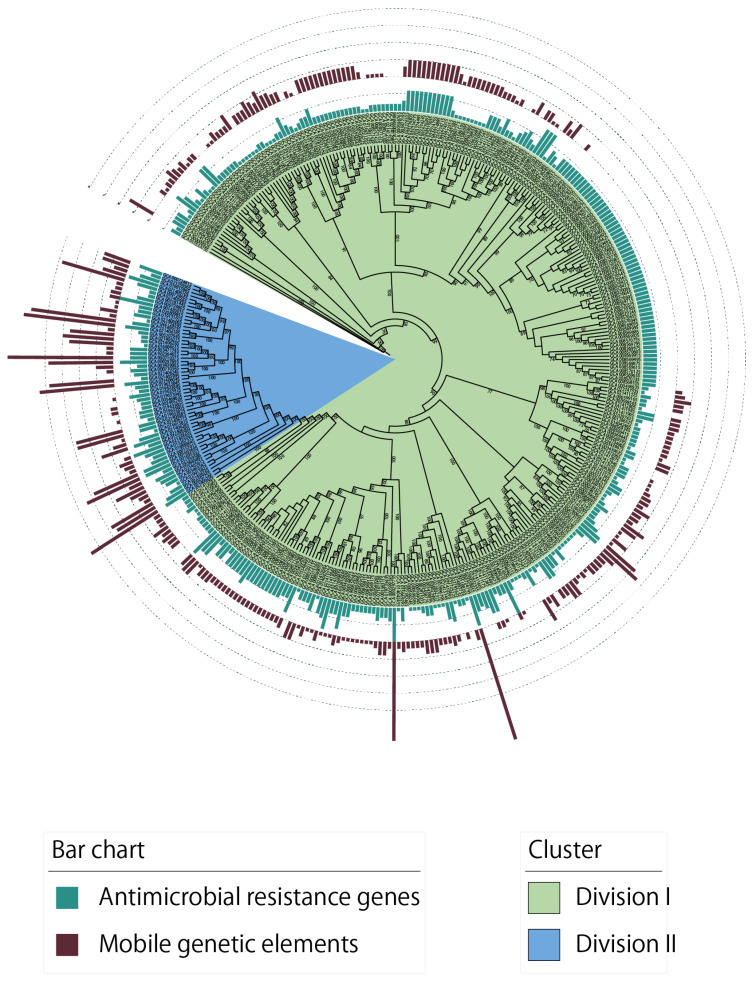
Phylogenetic relationship and antimicrobial resistance (AMR) gene and mobile genetic element (MGE) profiles of the 374 genomes of *B. fragilis*. The maximum likelihood cladogram was generated from concatenated core gene alignment and visualized using the Interactive Tree of Life program. Different background colors represent the two major clades (divisions). Bootstrap values are based on 1000 replications; only those >70% are indicated beside the branches. The green bar plots represent the number of identified AMR genes per genome and the brown bar plots represent the number of identified MGEs per genome.

A maximum likelihood (ML) tree of 374 *B. fragilis* strains, based on the alignment of the core genome sequences (Figure 2) revealed two basal clades, divisions I and II, as previously reported [8,17,44]. Whole-genome ANI analysis further supported this classification, dividing the genomes into two clades using a 95% ANI cutoff (Supplementary Figure S2). The type strain isolate was part of the larger division I (n = 317), whereas division II contained a considerably smaller number of members (n = 57) (Supplementary Table S1). Considering that the ANI values between the genomes of divisions I and II were below the threshold for the same species, division II likely represents distinct genomospecies [8,17,44]. EzBioCloud 16S rRNA identification service [30] revealed that the studied division II strains were most similar to *Bacteroides hominis* with >99% identity [45]. Furthermore, we observed that the reference genome of *B. hominis* L007 exhibits pair-wise ANI values greater than 98% with all the 57 division II strains (Supplementary Table S3). Therefore, *B. fragilis* division II should be considered a distinct species, *B. hominis*. However, both clades were included for a comparative evolutionary analysis to obtain insights into the diversification of this pathogen.

To assess the differences in gene content between genomes, a pan-genome network analysis was performed using the number of shared genes across a pair of genomes (Supplementary Figure S3). Genome-to-genome networks revealed two distinct modules that exhibited the same membership as divisions I and II in the core genome ML tree, indicating inter-clade gene content boundaries (Supplementary Table S4). Given the critical roles of AMR genes and MGEs in bacterial species’ evolution and diversification [46], we investigated their presence in individual genomes (Supplementary Tables S5 and S6). The number of AMR genes and MGEs in the genome of *B. fragilis* considerably differed, ranging from 1 to 10 AMR genes and 1 to 34 MGEs per genome (Figure 2 and Supplementary Table S7). Significant differences were noted between divisions I and II in the average AMR gene number (*p* < 0.001, Welch’s *t*-test) and MGEs (*p* < 0.001, Welch’s *t*-test) carried by individual genomes. The numbers of AMR genes and MGEs per genome were higher in division II than in division I. These data demonstrate that *B. fragilis* divisions I and II are genetically distinct lineages demonstrating divergence in their gene content, as evidenced by pan-genome divergence patterns [17,44].

### 3.4. Evolutionary Characteristics of the B. fragilis Pan-Genome

**Figure 3:**
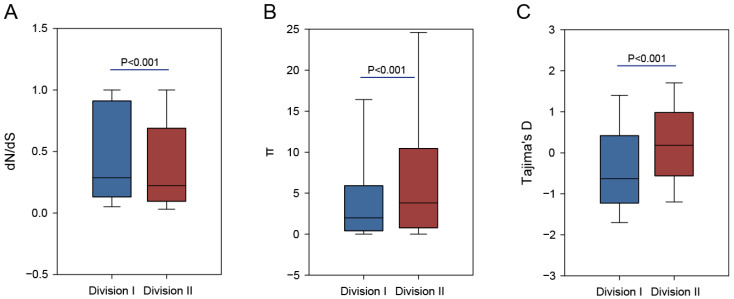
Evolutionary properties of the genes of division I and division II. (**A**) Box plot of the evolutionary rates (dN/dS ratio) for the genes of division I and division II. (**B**) Box plot of the nucleotide diversity (π) for the genes of division I and division II. (**C**) Box plot of the Tajima’s D-values for the genes of division I and division II. The Mann–Whitney U test was used to calculate the *p* values in (**A**–**C**).

To understand the evolutionary process behind *B. fragilis*’s genomic diversity, we investigated the genetic diversity and population dynamics within divisions I and II [47]. Initially, we examined the nonsynonymous/synonymous rate ratio (dN/dS) of the accessory and core gene clusters within both clades to measure differences in selective pressure. In general, the dN/dS ratio for the genes was <1 (Figure 3A), indicating that *B. fragilis*’s genome was subjected to purifying selection over time. The dN/dS ratio for division II genes was substantially lower than that of division I genes (Figure 3A), suggesting that the adaptive selection efficiency of division II genes is lower than that of division I genes.

The population genetic dynamics of divisions I and II genes demonstrated disparate patterns, as indicated by Tajima’s D and nucleotide diversity (π). The nucleotide diversity was lower for the division I population (Figure 3B), and the median Tajima’s D value was negative (Figure 3C). Contrastingly, division II population exhibited higher nucleotide diversity and a slightly positive median Tajima’s D value (Figure 3C). A positive Tajima’s D value indicates excessive intermediate-frequency polymorphisms, which is not expected under neutrality, suggesting a recent population contraction or balancing selection [48]. Contrastingly, a negative Tajima’s D value indicates excessive rare polymorphisms not expected under neutrality, likely caused by a recent population expansion or selective sweep [48]. Therefore, elevated Tajima’s D values observed in the division II population indicate that subpopulation contraction may have occurred following the recent *B. fragilis*’s species diversification. These differences in population genetics parameters between divisions I and II further highlight the barriers to gene flow between the clades, suggesting that they are genetically distinct in terms of pan-genome composition and nucleotide sequences.

### 3.5. Population Differentiation and Recombination of B. fragilis

**Figure 4:**
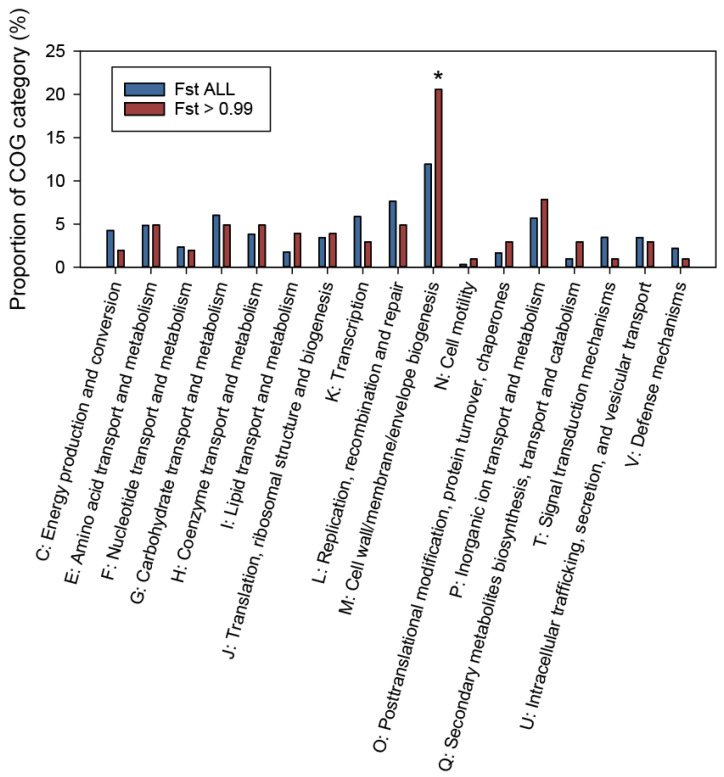
The Clusters of Orthologous Groups (COG) functional annotation of *B*. *fragilis* genes with Hudson’s fixation index (Fst) values > 0.99 (Fst > 0.99) and all the Fst-determined genes (Fst_all). Asterisks indicate significant enrichment of the Fst > 0.99 genes as compared to the Fst_all genes in the individual category (*p* < 0.05, Z-test). The COG functional categories without genes in the Fst > 0.99 category including “S: Function unknown” are not displayed.

We verified the extent of population differentiation between divisions I and II by examining gene-wise Fst values for pairs of gene sequences in each clade. The Fst values were determined for the genes that contained ≥5 sequences in each clade, including 5418 protein-coding genes. A histogram of the gene-wise Fst values demonstrated that the Fst distribution was markedly skewed towards a maximum value of 1 (Supplementary Figure S4). Given that the Fst values approaching 1 indicate complete structuring and fixation of genetic variability between populations, a considerable number of genes within the *B. fragilis* genome may have been highly differentiated or evolved between divisions I and II. Subsequently, we focused on the characteristics of the most highly differentiated genes, selecting 120 genes with Fst values > 0.99 (Supplementary Table S8). The high-Fst genes, which demonstrated near-complete differentiation, were annotated using the COG functional classification database (Figure 4). These genes included a higher gene proportion belonging to the COG categories like “I: Lipid transport and metabolism”, “M: Cell wall/membrane/envelope biogenesis”, and “P: Inorganic ion transport and metabolism”, as compared to all the Fst-determined genes. Notably, genes related to the COG category “M: Cell wall/membrane/envelope biogenesis” were the most significantly enriched in the higher Fst genes (*p* = 0.0109, Z-test). Genes responsible for the “M: Cell wall/membrane/envelope biogenesis” are critical in determining the efficiency and specificity of gene transfer between bacteria [43]. Therefore, higher Fst genes with these cell-surface functions may play a crucial role in population differentiation.

**Figure 5:**
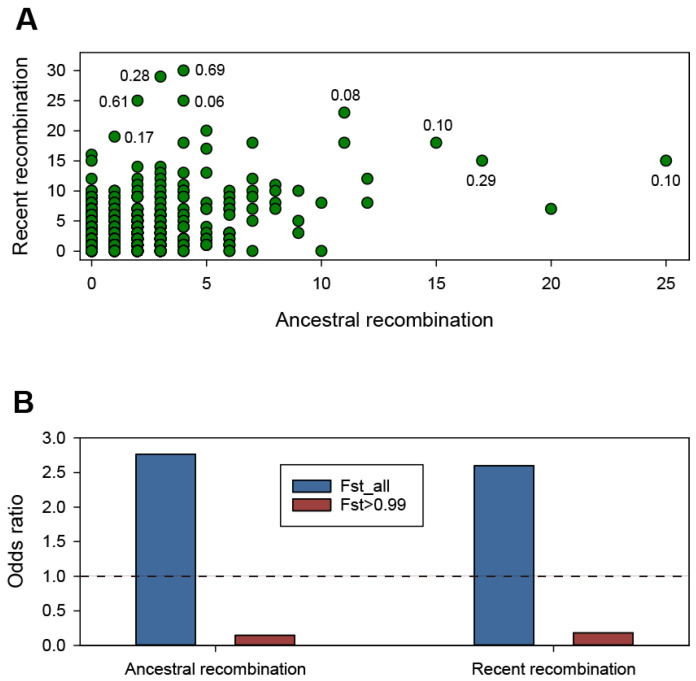
Heterogeneity in the recombination frequency in the *B*. *fragilis* genomes. (**A**) Genes that have undergone recent and ancestral recombination. Horizontal and vertical axes indicate the estimated number of ancestral recombinations and the estimated number of recent recombinations, respectively. The Hudson’s fixation index (Fst) values of some of the most frequently recombined genes are indicated beside the plot. (**B**) The odds ratio of recombinations versus non-recombinations for Fst > 0.99 and Fst_all genes. Ancestral recombination and recent recombination are separately evaluated and are illustrated in the figure. An odds ratio > 1 and <1 indicate that the genes are enriched with recombinations and a deficiency in recombination events within the genes, respectively.

Recombination is crucial for the evolution of several bacterial species, facilitating the emergence of novel phenotypes and their diversification into new lineages [49]. We identified frequently recombining genes in the *B. fragilis* pan-genome by running fastGEAR [42] on individual sequence alignments of the core and shared accessory genes. Of the 18,521 genes that comprised the pan-genome gene clusters occurring in ≥5 strains, 2923 and 995 genes were involved in recent and ancestral recombinations, respectively (Figure 5A, Supplementary Table S9). Although several of the frequently recombined genes have hypothetical or unknown functions according to Prokka annotations, some of the most frequently recombined genes are suggested to be related to gene transfer and protection from invading DNA according to the annotation of the eggNOG database (Supplementary Table S9). We mapped Fst values to the recombination frequency of the genes to further document the relationship between recombination frequency and population differentiation. Genes with a high recombination degree tended to have relatively low Fst values (Figure 5A, Supplementary Table S9). Furthermore, a comparison between genes with Fst values > 0.99 (Fst > 0.99) and all Fst-determined genes (Fst_all) demonstrated a significant depletion of ancestral recombination in the Fst > 0.99 genes (Figure 5B; *p* = 0.02252, Fisher’s exact test). A comparable trend was observed for recent recombination in the Fst > 0.99 genes (Figure 5B; *p* = 2.363 × 10^−5^, Fisher’s exact test). Thus, most genes with the highest Fst values were markedly suppressed for recombination. Elevated recombination rates have been shown to facilitate adaptation in bacterial populations [50]. However, this process can impede the divergence of discrete sublineages, resulting in the formation of genetically interchangeable populations within a species [51]. Therefore, the highest Fst genes with minimal recombination observed in the *B. fragilis* pan-genome could be those related to population differentiation across divisions I and II, ultimately resulting in the emergence of two distinct genomospecies and contributing to the speciation process [52].

## 4. Conclusions

In this study, we analyzed the genomic architecture and evolutionary patterns of *B. fragilis* through pan-genome analysis. Our results indicated that *B. fragilis* exhibits an open pan-genome, signifying substantial variability in the accessory genome with the addition of more sequenced genomes. COG category analysis of the accessory genome revealed considerable enrichment in “L: Replication, recombination, and repair”, highlighting the roles of these functions in gene transfer and diversification.

Phylogenetic analysis of the *B. fragilis* core genome sequences confirmed the separation of the population into two genomospecies, designated divisions I and II as previously reported. This separation was further supported by a pan-genome network analysis based on shared genes across genomes. Exploration of the population genetic dynamics of divisions I and II demonstrated separation between the two divisions at the level of genetic diversity, indicating barriers to gene flow. Notably, genes from division II demonstrated higher Tajima’s D values, suggesting that the separation of division II occurred after the recent species diversification of *B. fragilis*. Average nucleotide identity and 16S rRNA analyses showed that *B. fragilis* division II represents a distinct species, *B. hominis*.

The distribution of gene-wise Fst values for the pair of gene sequences in each division shifted to an extreme value of 1, suggesting that several genes within the *B. fragilis* genome are highly differentiated or evolved between divisions I and II. Furthermore, a comparison between genes with Fst values > 0.99 and all Fst-determined genes revealed a substantial recombination depletion in genes with Fst values > 0.99. Consequently, most genes with the highest Fst values were markedly suppressed for recombination. Thus, the highest Fst genes with minimal recombination may serve as the basis for differentiation between the two genomospecies, divisions I and II. In summary, our comparative evolutionary genomics study highlighted distinct genetic signatures between *B. fragilis* divisions I and II, providing insights into their evolutionary characteristics and speciation processes.

## Data Availability

The original contributions presented in the study are included in the article/Supplementary Materials, further inquiries can be directed to the corresponding author.

## Supplementary Materials

The following supporting information can be downloaded at: https://www.mdpi.com/article/10.3390/genes15121519/s1, Figure S1: The number of new genes with an increase in the number of *Bacteroides fragilis* genomes; Figure S2: Heatmap of the pairwise genome-wide average nucleotide identity (ANI) values of the *Bacteroides fragilis* strains used in this study; Figure S3: Pan-genome network based on the gene presence/absence matrix; Figure S4: Distribution of gene-wise Hudson’s fixation index (Fst) values for the pair of gene sequences in divisions I and II; Table S1: *Bacteroides fragilis* genomes used in this study; Table S2: The list of the genes considered as core, soft core, shell, and cloud categories; Table S3: ANI value results of each strain of division II against the genomes of both reference strains (*B. hominis* L007 and *B. fragilis* NCTC 9343); Table S4: The presence/absence gene frequencies for divisions I and II; Table S5: The type and distribution of AMR genes between the genomes of divisions I and II; Table S6: The type and distribution of MGEs between the genomes of divisions I and II; Table S7: Antimicrobial resistance genes and mobile genetic elements in the *Bacteroides fragilis* genomes; Table S8: Properties of highly differentiated genes with Hudson’s fixation index (Fst) values of >0.99; Table S9: Properties of the frequently recombining genes in the *Bacteroides fragilis* pan-genome.
