# Supplementary material for: Comparative Evolutionary Genomics Reveals Genetic Diversity and Differentiation in Bacteroides fragilis
Source: Genes (Basel). 2024 Nov 27;15(12):1519. doi: 10.3390/genes15121519 (PMC11675351; doi:10.3390/genes15121519)
Supplement: Supplementary file 1 [file genes-15-01519-s001.zip › Legends for Supplementary files.pdf]

Legends for Supplementary files

Figure S1
The number of new genes with an increase in the number of *Bacteroides fragilis* genomes.

Figure S2
Heatmap of the pairwise genome-wide average nucleotide identity (ANI) values of the *Bacteroides fragilis* strains used in this study. The ANI values calculated using the pyani tool are color-coded according to the provided scale bar.

Figure S3
Pan-genome network based on the gene presence/absence matrix. The nodes and edges represent individual strains and similarity coefficients based on the number of shared genes, respectively.

Figure S4
Distribution of gene-wise Hudson's fixation index (Fst) values for the pair of gene sequences in divisions I and II.

Table S1
Bacteroides fragilis genomes used in this study.

Table S2
The list of the genes considered as core, soft core, shell and cloud categories.

Table S3
ANI value results of each strain of division II against the genomes of both reference strains (*B. hominis* L007 and *B. fragilis* NCTC 9343).

Table S4
The presence/absence gene frequencies for divisions I and II.

Table S5
The type and distribution of AMR genes between the genomes of divisions I and II.

Table S6
The type and distribution of MGEs between the genomes of divisions I and II.

Table S7
Antimicrobial resistance genes and mobile genetic elements in the *Bacteroides fragilis* genomes.

Table S8

Properties of highly differentiated genes with Hudson's fixation index (Fst) values of > 0.99.

Table S9

Properties of the frequently recombining genes in the *Bacteroides fragilis* pangenome.
