# Supplementary figures and images for: Comparative Evolutionary Genomics Reveals Genetic Diversity and Differentiation in Bacteroides fragilis
Source: Genes (Basel). 2024 Nov 27;15(12):1519. doi: 10.3390/genes15121519 (PMC11675351; doi:10.3390/genes15121519)

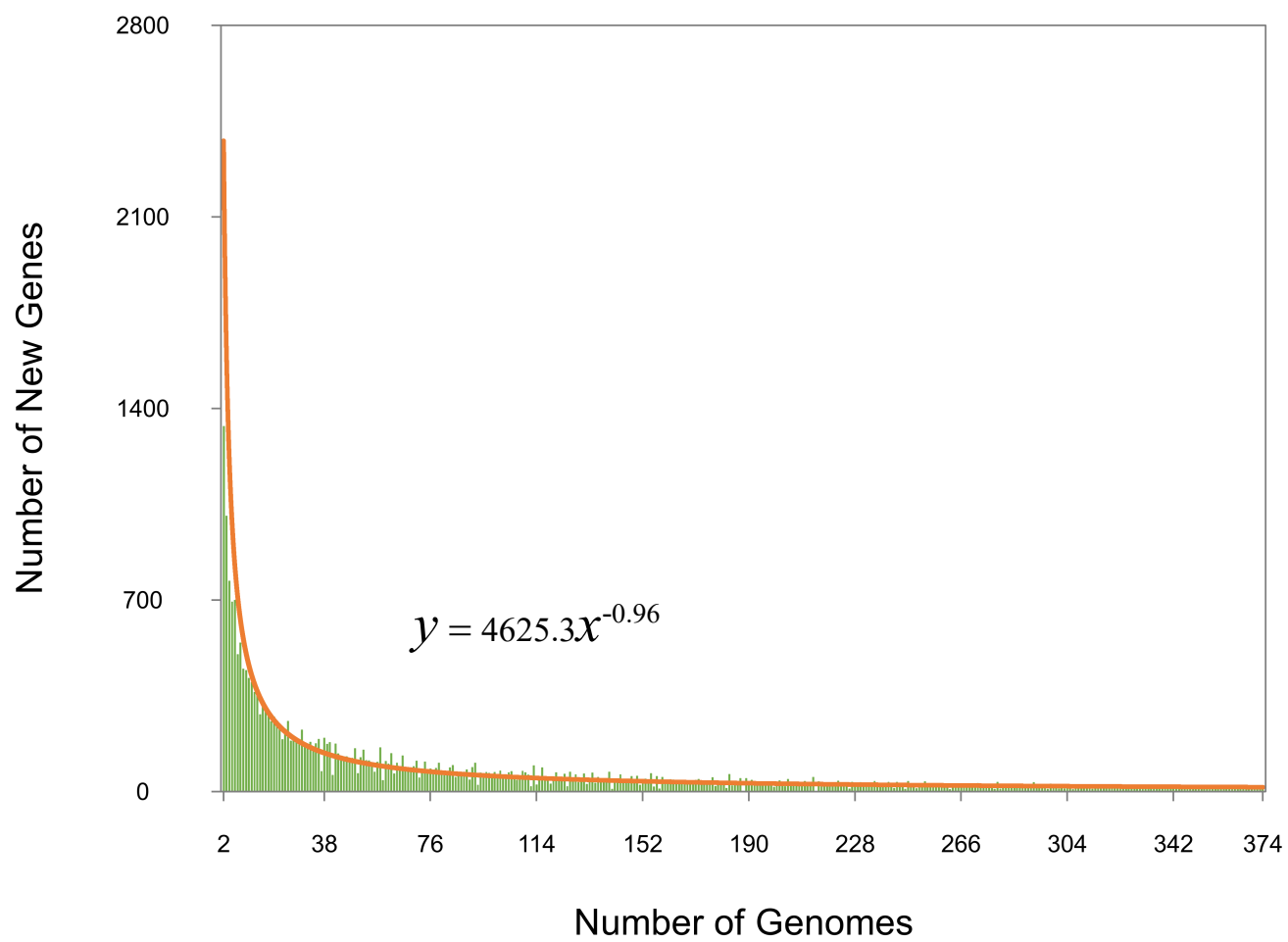

Supplement: Supplementary file 1 [file genes-15-01519-s001.zip › Figure S1.pdf]

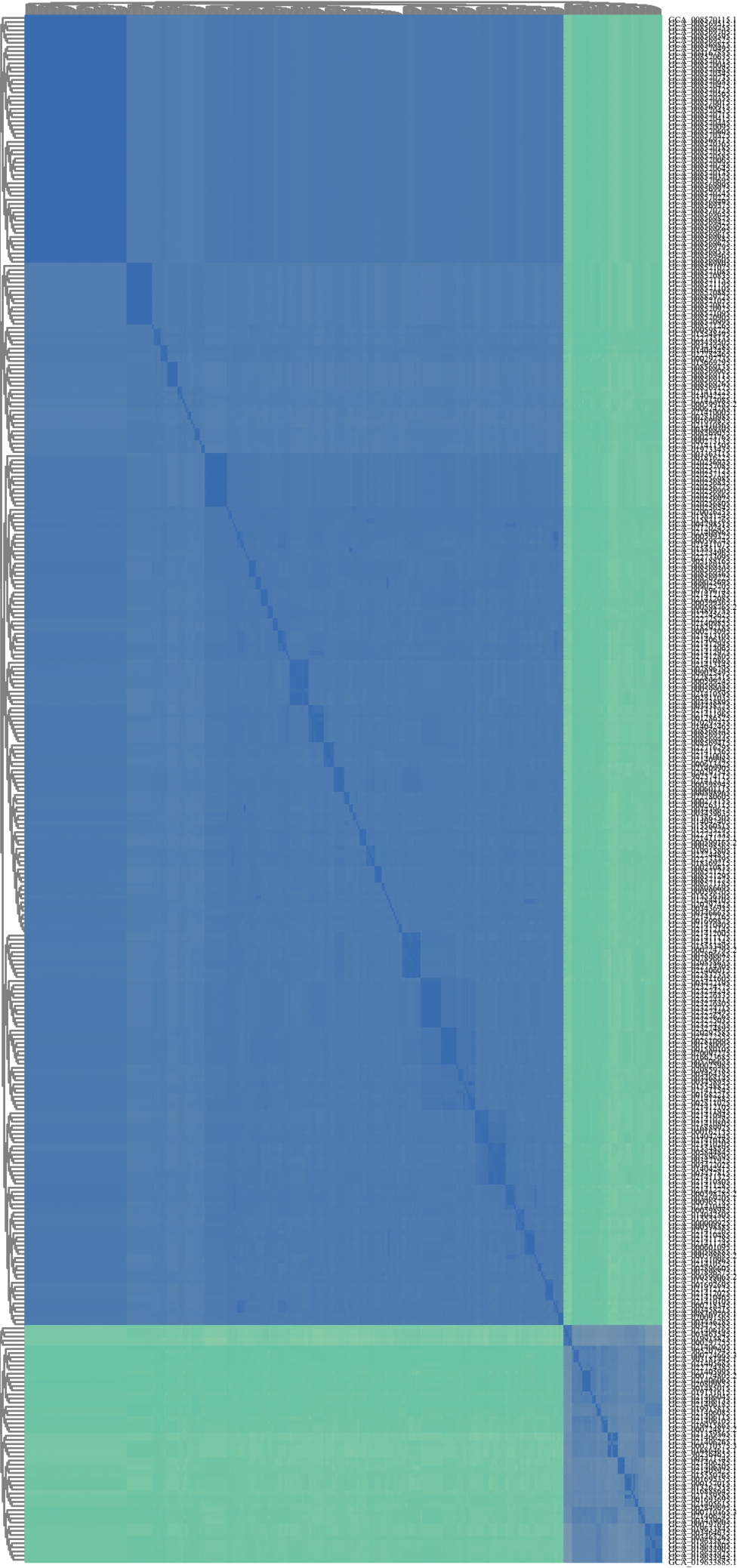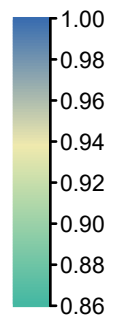

Supplement: Supplementary file 1 [file genes-15-01519-s001.zip › Figure S2.pdf]

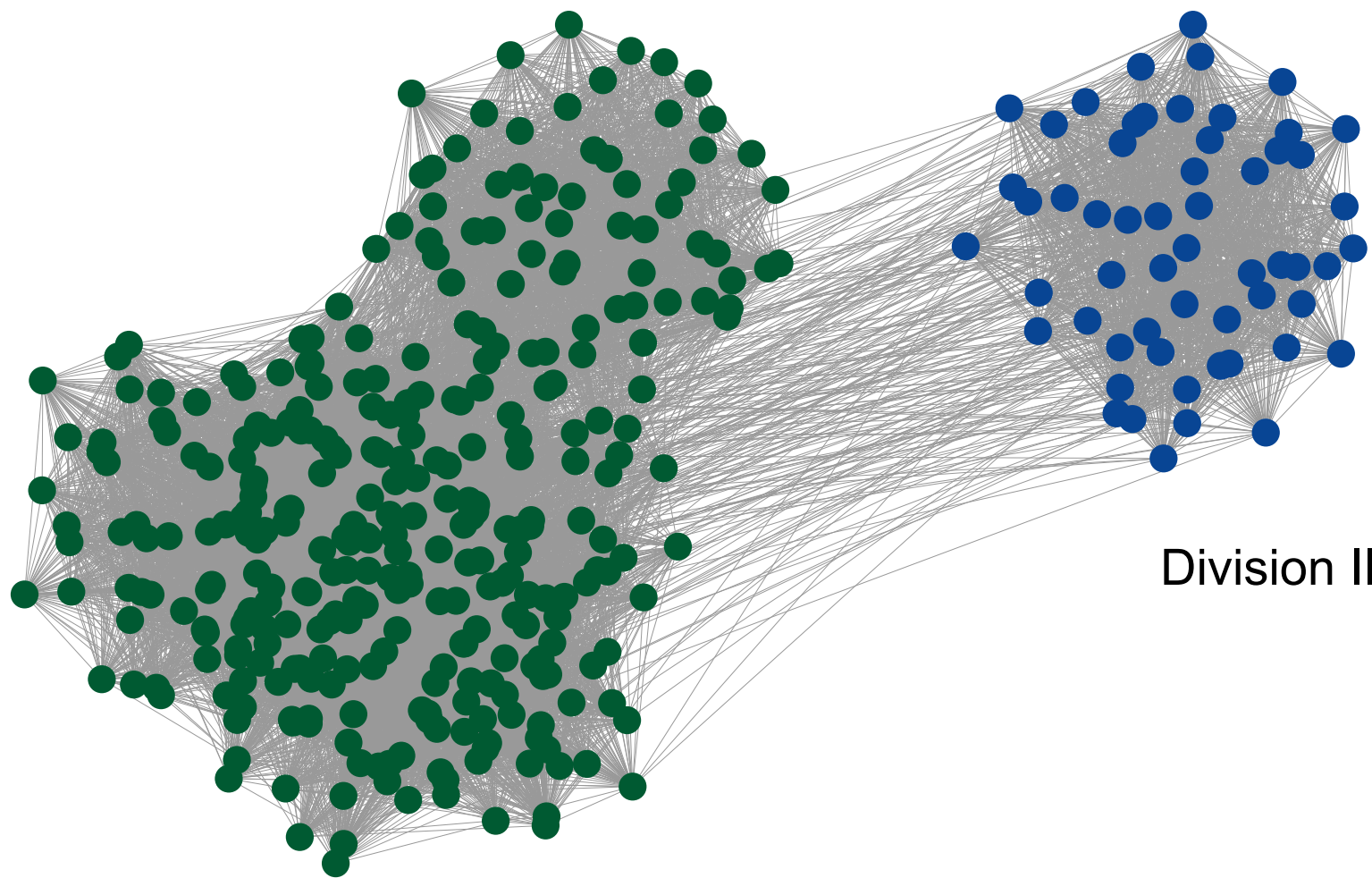

Division I

Division II

Supplement: Supplementary file 1 [file genes-15-01519-s001.zip › Figure S3.pdf]

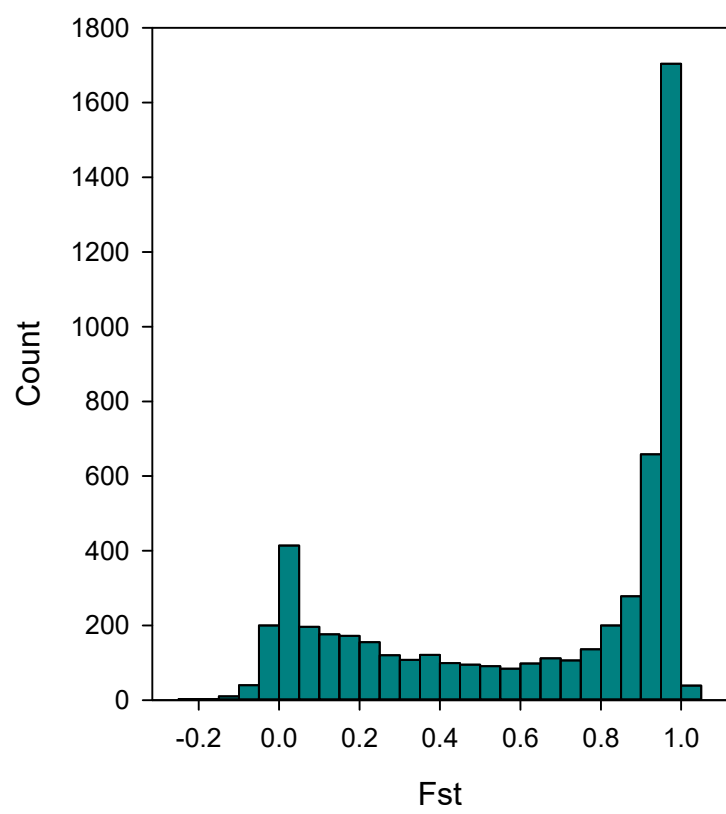

Supplement: Supplementary file 1 [file genes-15-01519-s001.zip › Figure S4.pdf]
